# Sonic hedgehog and Wnt: antagonists in morphogenesis but collaborators in axon guidance

**DOI:** 10.3389/fncel.2013.00086

**Published:** 2013-06-10

**Authors:** Evelyn C. Avilés, Nicole H. Wilson, Esther T. Stoeckli

**Affiliations:** Institute of Molecular Life Sciences, University of ZurichZurich, Switzerland

**Keywords:** neural circuit, Frizzled, Ryk, Smoothened, spinal cord, morphogen, attraction, repulsion

## Abstract

As indicated by their name, morphogens were first identified for their role in the formation of tissues early in development. Secreted from a source, they spread through the tissue to form gradients by which they affect the differentiation of precursor cells in a concentration-dependent manner. In this context, the antagonistic roles of the morphogens of the Wnt family and Sonic hedgehog (Shh) in the specification of cell types along the dorso-ventral axis of the neural tube have been studied in detail. However, more recently, morphogens have been demonstrated to act well beyond the early stages of nervous system development, as additional roles of morphogen gradients in vertebrate neural circuit formation have been identified. Both Wnt and Shh affect neural circuit formation at several stages by their influence on neurite extension, axon pathfinding and synapse formation. In this review, we will summarize the mechanisms of morphogen function during axon guidance in the vertebrate nervous system.

## Introduction

Morphogens, defined as secreted molecules that act in a concentration gradient to affect the differentiation of precursor cells, are also involved in the establishment of neural connections. Both Sonic hedgehog (Shh) and Wnts play important roles in different cellular events during neural development (Wilson and Stoeckli, 2012). Shh activity is triggered by its binding to the Patched (Ptc) receptor and the consequent derepression of Smoothened (Smo), leading to translocation of GliA to the nucleus, inducing the transcription of target genes. Wnts regulate transcriptional activity by binding to a receptor complex formed by a Frizzled (Fz) family member and Lrp5/6. This in turn leads to the inhibition of GSK3β and the accumulation of β-catenin, which can enter the nucleus and act together with Tcf/Lef transcription factors to regulate target gene expression. In addition to this so-called canonical pathway, Wnt ligands are able to activate alternative signaling pathways, such as the planar cell polarity (PCP) pathway and the calcium pathway (see Section Molecular mechanisms of Wnt-mediated axon guidance). The PCP pathway is not only involved in tissue polarity but also affects axon guidance and cell migration.

**Figure 1:**
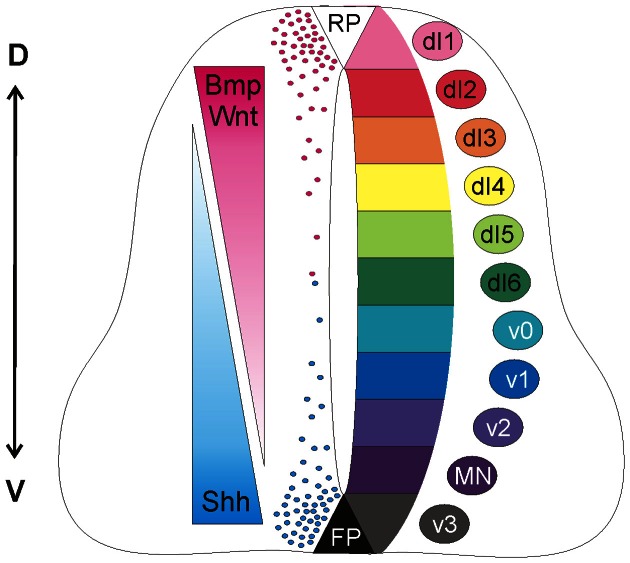
**Antagonistic activities of Shh and Wnt/BMP pattern the developing spinal cord**. Counteracting gradients of Shh, secreted from the floorplate (FP), and Wnt/BMP, derived from the roof plate (RP) induce the concentration-dependent differentiation of precursor cells along the dorso-ventral (D-V) axis. During morphogenesis, Shh and Wnts have antagonistic functions. Shh promotes the formation of Gli activator forms, while Wnts directly induce the expression of Gli3, which acts as a transcriptional repressor in the absence of Shh (see Dessaud et al., 2008, for details). In turn, the specific combinations of transcription factors induced by Shh and Wnts generate a cell identity code that specifies the neural progenitor subtypes. As these cells exit the cell cycle, they distribute laterally in a specific order along the dorso-ventral axis (dI1-v3).

During morphogenesis, Shh and Wnts act antagonistically in the patterning of the neural tube (Figure 1). Shh has a ventralizing activity (Dessaud et al., 2008), which is antagonized by Wnt signaling (Ulloa and Martí, 2010). At the molecular level, Gli3, a signaling molecule downstream of Shh, appears to be the link, as Gli3 expression is induced by Wnts (Ulloa and Martí, 2010).

Roles for Shh and Wnts later in neural development have been described more recently. After an initial report on Wnt's role in commissure formation in *Drosophila* (Yoshikawa et al., 2003), Wnts were also implicated in axon guidance in vertebrates (Lyuksyutova et al., 2003; see below). At about the same time, Shh was shown to be involved in vertebrate axon guidance (Charron et al., 2003; Bourikas et al., 2005; see below). So far, such a role for Shh has not been found in invertebrates. In addition, both Wnts and Shh were found to affect synaptogenesis (Salinas and Zou, 2008; Harwell et al., 2012). Morphogen signaling in these late stages of neural development is relatively poorly understood, as it is more complex than canonical signaling.

In this review, we will discuss the role of morphogens in neural circuit formation by concentrating on axon guidance. In many areas of the developing nervous system, Wnts and Shh are expressed in overlapping areas. In contrast to their effects in early development, where they were found to antagonize each other, Shh and Wnts often collaborate in axon guidance, although the effect on a navigating growth cone may still be antagonistic. Some of the molecular mechanisms underlying these signaling activities are beginning to be elucidated.

## Shh and Wnts contribute to axon guidance of many different neuronal populations

### dI1 commissural axons of the spinal cord

**Figure 2:**
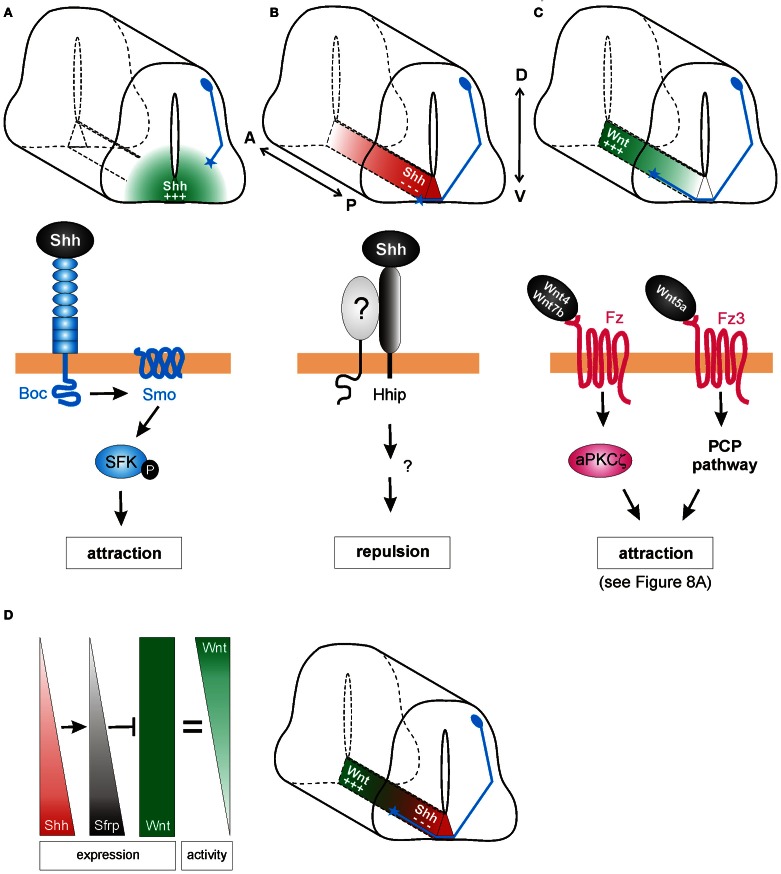
**Shh and Wnts guide commissural axons in the vertebrate spinal cord. (A)** Pre-crossing commissural axons (blue) are attracted ventrally toward the midline by an increasing gradient of Shh produced in the floorplate (green). The attractive effect of Shh is mediated by Smoothened (Smo) and Brother of CDO (Boc) in a transcription-independent manner. Instead, the activation of Src family kinases (SFK) induces cytoskeletal rearrangements in the growth cone. **(B)** The response of commissural axons to Shh switches from attraction to repulsion when axons reach the midline. Post-crossing commissural axons are pushed anteriorly by a posterior^high^ to anterior^low^ gradient of Shh (red). The repellent activity of Shh is mediated by Hedgehog-interacting protein (Hhip), a receptor that is transiently upregulated on commissural axons at the time of their turning into the longitudinal axis. An additional signaling co-receptor may also be involved. **(C)** An anterior^high^ to posterior^low^ gradient of Wnt activity works in parallel to Shh repulsion to attract post-crossing commissural axons anteriorly. Depending on the species, Wnt4, Wnt5a, and Wnt7a are attractants for post-crossing commissural axons via non-canonical pathways. In mouse, Fz3, in response to Wnt4/Wnt7b, activates a complex containing an atypical protein kinase C (aPKCζ). In response to Wnt5a, the PCP pathway is activated. See text for more details. **(D)** In chick, Shh was shown to shape Wnt activity indirectly. Wnt5a and Wnt7a are expressed uniformly along the longitudinal axis. In addition to its direct effect on post-crossing commissural axons, Shh induces the expression of the Wnt antagonist Sfrp1 in a posterior^high^ to anterior^low^ gradient in the floorplate. The antagonistic activity of Sfrp in turn regulates the activity of Wnts in the floorplate, such that an attractive “activity gradient” of Wnt is formed. Thus, Shh (red) and Wnt (green) gradients collaborate to guide post-crossing commissural axons anteriorly.

Commissural axons in the developing spinal cord have provided an accessible, informative *in vivo* model to investigate the molecular mechanisms of axon guidance (Chédotal, 2011). During development, dorsally-located dI1 commissural neurons project their axons ventrally toward and across the ventral midline at the floorplate, forming axon commissures that enable bilateral neural communication (Figure 2). After crossing the midline, the axons make an abrupt 90° turn and extend rostrally in close contact with the contralateral floorplate border. Despite the apparent simplicity of this trajectory, the navigating axons must make many complex pathfinding decisions *en route* to their target. Initially, they perceive the floorplate as attractive, but upon arrival at this intermediate target, they must switch their response to repulsion in order to move on. Upon exiting the floorplate, dI1 axons make a sharp rostral turn into the longitudinal axis to continue their post-crossing trajectory. As an intermediate target for these axons, the floorplate is a major source of attractive and repulsive, long-range and short-range cues. The correct interpretation of these signals by the navigating axons is only possible by precise spatiotemporal control over cellular signaling pathways (Stein and Tessier-Lavigne, 2001; Zisman et al., 2007; Yoon et al., 2009).

The initial ventral projection of commissural axons is determined in part by Netrin1, a long-range, floorplate-derived chemoattractant (Serafini et al., 1996). Commissural axons express the Netrin1 receptor DCC (deleted in colon cancer) (Keino-Masu et al., 1996), and accordingly, mice mutant for either *Netrin1* or *DCC* display severe axon guidance defects, in which many commissural axons fail to invade the ventral spinal cord and are unable to cross the midline (Fazeli et al., 1997). However, not all commissural axons are affected in *Netrin1* mutant mice, suggesting that an additional chemoattractive mechanism acts in parallel to Netrin1-DCC to guide axons toward the floorplate.

Indeed, Charron et al. (2003) demonstrated that in *Netrin*-deficient mice Shh attracts commissural axons ventrally (Figure 2A). *In vitro*, the *Netrin1*-deficient floorplate was still able to elicit commissural growth cone turning, an effect that was mimicked by the presence of Shh-expressing COS cells. Shh also acted directly as a chemoattractant on isolated *Xenopus* spinal axons. Thus, Shh is a chemoattractant for commissural axons in collaboration with Netrin-1, although its effect is normally masked by the stronger effect of Netrin-1. When Smo was inhibited by cyclopamine, or when Smo was conditionally inactivated in the dorsal spinal cord, the chemoattractant effects of Shh were blocked *in vitro* and commissural axons projected abnormally toward the floorplate *in vivo*. Thus, Shh acts via Smo as an axonal chemoattractant in the dorsoventral axis of the spinal cord.

Although the attractive guidance effects of Shh were transduced via Smo, Smo does not bind Shh directly. Thus, the next step was to identify the cell surface receptor/s responsible for Shh binding. Two structurally related candidates, Cdon (Cell adhesion molecule-related/downregulated by Oncogenes) and Boc (Brother of Cdon) were tested for their ability to bind Shh and mediate its attractive axon guidance effects (Okada et al., 2006). While both receptors bound specifically to Shh, only Boc was expressed by differentiating commissural neurons. In Boc knockout mice, commissural axons were misdirected and invaded the motor columns as they approached the floorplate, a phenotype which resembled that previously described following disruption of Smo. Additionally, RNA-interference-mediated knockdown of Boc impaired the turning response of rat commissural axons toward an ectopic source of Shh *in vitro*. Taken together, these data suggested that Boc was an essential receptor for Shh in attractive commissural axon guidance.

In addition to its role as an attractant of pre-crossing axons toward the floorplate in the dorsoventral axis (Charron et al., 2003), Shh also acts as a repulsive guidance cue for post-crossing axons, directing them into the longitudinal axis toward the brain (Bourikas et al., 2005; Yam et al., 2012). In chicken, this rapid switch from attractant to repellant is due to a change in the growth cone receptors that detect and transduce the Shh signal (Figure 2B). When Shh was reduced in a spatiotemporally-controlled manner by *in ovo* electroporation of dsRNA, post-crossing commissural axons either stalled at the floorplate exit site, or even turned caudally instead of rostrally. Consistent with its decreasing posterior^high^ to anterior^low^ expression pattern in the chick floorplate, Shh can directly repel post-crossing commissural axons from spinal cord explants (Bourikas et al., 2005). In line with the finding that neither Smo nor Ptc mRNAs were expressed in commissural neurons after their axons reached the midline, Hedgehog-interacting protein (Hhip) was identified as the Shh receptor mediating repulsion. Hhip was transiently upregulated at the time when the commissural axons turn into the longitudinal axis (Bourikas et al., 2005). A recent report confirms that Shh also acts as a longitudinal repellent in mammals (Yam et al., 2012), although the role of Hhip could not be confirmed in the mouse.

Experiments with mouse spinal cord explants from the Zou laboratory also implicated Wnt ligands in post-crossing commissural axon guidance (Lyuksyutova et al., 2003). According to the anterior^high^ to posterior^low^ gradient of *Wnt4* mRNA expression in the floorplate along the mouse spinal cord, Wnt4 was shown to attract post-crossing commissural axons (Figure 2C). Mice lacking the Wnt receptor Frizzled3 (Fz3) exhibited randomization of commissural axons after crossing the floorplate, suggesting that Wnt4 might act via the Fz3 receptor to attract these axons (Lyuksyutova et al., 2003). However, the genetic or biochemical interaction between Wnt4 and Fz3 has, to date, not been shown. More recently, post-crossing commissural axons were shown to respond to a Wnt activity gradient *in vivo*, in the chicken spinal cord (Domanitskaya et al., 2010). In the chicken, Wnt5a and Wnt7a were shown to act as attractive cues *in vivo*, as assessed by *in ovo* RNA interference. In contrast to the mouse, *Wnt5a* and *Wnt7a* are not expressed in a gradient along the A-P axis of the lumbar spinal cord in chicken. Rather, a Wnt activity gradient (anterior^high^ to posterior^low^) is shaped by the graded expression (anterior^low^ to posterior^high^) of the Wnt antagonist, Secreted frizzled-related protein (Sfrp) (Figure 2D; Domanitskaya et al., 2010).

Intriguingly, Sfrp is a transcriptional target of Shh not only in mesodermal tissue (Lee et al., 2000) but also in the developing neural tube. Ectopic expression of Shh in the chicken spinal cord induced both *Sfrp1* and *Sfrp2* (Domanitskaya et al., 2010), suggesting that during normal development, the graded expression (anterior^low^ to posterior^high^) of *Sfrps* in the floorplate was induced by the corresponding gradient of Shh. Interestingly, these Sfrps did not act directly as guidance cues, but rather indirectly by preventing Wnts from binding to their Fz receptors and thus by shaping a Wnt activity gradient. Together, these findings identified a close link between the two guidance cues for post-crossing commissural axons in chicken: Shh not only repels post-crossing commissural axons directly (Bourikas et al., 2005), but also influences Wnt activity indirectly by inducing the graded expression of the Wnt antagonists Sfrp1 and Sfrp2, which in turn shape the functional gradient of Wnt activity (Figure 2D). Whether Shh induces Sfrps in the mouse floorplate (a mechanism that could sharpen the existing Wnt4 expression gradient) is unknown. The molecular pathway by which Shh induces Sfrps also requires investigation, as do the signaling mechanisms downstream of Wnt in post-crossing commissural axon guidance in the chicken.

### Retinal ganglion cell axons and topographic mapping

**Figure 3:**
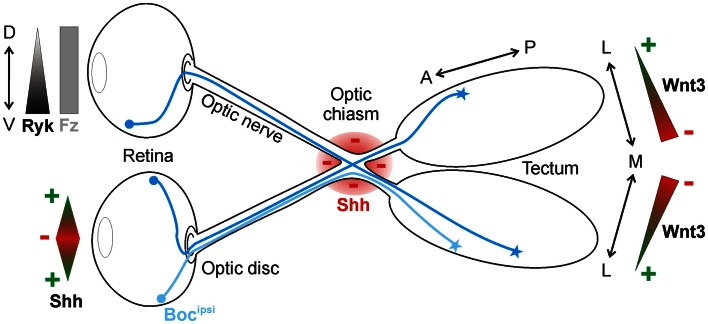
**Shh and Wnt3 are axon guidance cues in the visual system**. In the retina, Shh is bifunctional: low concentrations promote RGC axon outgrowth toward the optic disc, whereas high concentrations push axons into the optic nerve. Shh is also expressed along the border of the optic chiasm (red), where it acts as a repellant for RGC axons in the optic nerve. Ipsilaterally projecting RGC axons express Boc (Boc^ipsi^), which mediates Shh repulsion at the chiasm. Wnt3 is expressed in a medial^high^-to-lateral^low^ gradient in the tectum. At high concentrations, Wnt3 inhibits the growth of both dorsal and ventral RGC axons via Ryk as a receptor. At low concentrations, Wnt3 stimulates the growth of dorsal RGC axons to the lateral tectum in a Fz-dependent manner.

Another well-studied model for neural circuit formation is the visual system (Erskine and Herrera, 2007; Petros et al., 2008). During development, retinal ganglion cells (RGCs) of the eye project their axons through quite diverse microenvironments *en route* to their targets in the brain. Initially, RGC axons all grow to the optic disc at the center of the retina, where they exit the eye and course within the optic nerve toward the midline, where they must decide to grow either ipsilaterally or contralaterally. By crossing the midline, RGC axons form the optic chiasm. Post-chiasmic RGC axons then travel along the lateral surface of the neuroepithelium in the diencephalon, forming the optic tract. Finally, the axons turn caudally toward their final target, the optic tectum. Growing evidence indicates that Shh is an important guidance cue for RGC axons at several stages along this route. Interestingly, Shh displays a dual activity in this system. Depending on its concentration, Shh either promotes or prevents RGC axon growth (Kolpak et al., 2005, 2009; Gordon et al., 2010). Upon reaching the tectum, RGC axons rely on Wnt signaling in order to correctly identify their targets (Schmitt et al., 2006) (Figure 3).

The initial guidance of RGC axons within the retina toward the optic disc is regulated by several classical cues, including Slit1 (Jin et al., 2003), Netrin1 (Deiner et al., 1997), and EphBs (Birgbauer et al., 2000). Shh acts in addition to these cues to influence RGC axon projection inside the retina. Kolpak et al. (2005) identified Shh as a factor secreted from the central retina that positively affected RGC axon growth. When RGC axons were exposed to low concentrations of Shh *in vitro*, they displayed increased outgrowth. This effect was blocked by the addition of cyclopamine, suggesting that Shh promoted axon outgrowth via Smo. When faced with a choice of substrate in stripe assay experiments, RGC axons preferred to grow on stripes containing a low concentration of Shh. These positive outgrowth responses to Shh occurred rapidly, suggesting that Shh was acting directly on the growth cone, via a transcription-independent pathway.

In the same study, Kolpak et al. (2005) found that high concentrations of Shh instead had negative outgrowth effects on RGC axons, as assessed in their co-culture and stripe assay experiments. Like the positive effects, the concentration-dependent negative effects of Shh were mediated by Smo, since they were blocked by cyclopamine. Taken together, the experiments suggested that the precise level of Shh protein expression inside the retina is critical for the projection of RGC axons toward the optic disc.

Shh has also been reported as a repellant molecule for RGCs further along in their trajectory. After leaving the eye through the optic disc, RGC axons approach the optic chiasm and prepare to innervate either the ipsilateral or contralateral side of the brain. Shh is expressed along the border of the optic chiasm, defining a barrier at the ventral midline that could guide the projection of RGC axons (Figure 3). In agreement with this idea, ectopic expression of Shh at the midline prevents RGC axons from crossing (Trousse et al., 2001). Conversely, the injection of E13.5 mouse embryos with a hybridoma producing a Shh-blocking antibody causes aberrant projection of RGC axons at the optic chiasm by E18.5 (Sanchez-Camacho and Bovolenta, 2008). However, in that study, the blockade of Shh signaling for five days could have led to changes in midline patterning, leaving open the possibility that the effects observed were not due to a direct guidance effect of Shh but rather to patterning defects. *In vitro* experiments do however support the notion that Shh acts directly as a chemorepellant for RGC axons, since axons from retinal explants were reduced in number and length following the addition of exogenous recombinant Shh. Time-lapse analysis revealed that axons from retinal explants rapidly retracted in the presence of Shh, in a concentration-dependent manner (Kolpak et al., 2005).

A recent report suggests that Boc is the receptor responsible for the repulsion of ipsilateral RGC axons from Shh at the chiasm (Fabre et al., 2010). Interestingly, this finding indicates that Boc-mediated transduction of the Shh signal leads to opposite axon guidance effects in commissural neurons (attraction; see Section dI1 commissural axons of the spinal cord) versus RGC neurons (repulsion). The molecular mechanisms underlying these differential, Boc-mediated effects of Shh are yet to be elucidated. Additionally, Smo has also been implicated in the guidance of RGC axons *in vivo* (Sanchez-Camacho and Bovolenta, 2008). When a Smo inhibitor was electroporated into contralaterally-projecting RGC neurons, the axons displayed growth and guidance defects at the midline. These experiments suggested that Shh signaling functions cell-autonomously to control the pathfinding of RGC axons (Sanchez-Camacho and Bovolenta, 2008). However, Boc is not expressed in the contralaterally-projecting RGCs (Fabre et al., 2010), thus the identity of the Shh receptor mediating this effect of Shh on contralateral RGC axons remains to be determined. It is also unknown whether the ipsilaterally-projecting RGC axons require Smo for their response to Shh.

In *Xenopus* embryos, Shh has also been demonstrated to guide RGC axons within the optic tract, after they have crossed the midline (Gordon et al., 2010). *Xshh* was expressed adjacent to the ventral optic tract during RGC axon extension, and the RGCs were found to co-express Ptc and Smo. Bath application of cyclopamine caused abnormal phenotypes, including defasciculation and widening of the ventral optic tract, axonal misprojection into the neuroepithelium and targeting errors in the tectum. Conversely, ectopic activation of Shh signaling by implanting beads soaked in N-Shh caused a deflection of RGC axons away from their normal pathway, or a cessation of axonal extension. Together, these findings indicate that Shh signaling is required to define the path of retinal axons in the optic tract.

Since *Xshh* mRNA expression was not detected near the optic tectum, it is not clear how Shh could be influencing axonal targeting in the tectum directly. Shh was suggested to either diffuse over long distances from ventrally located sources (Gordon et al., 2010) or it may come from the RGC axons themselves. RGC axons have been reported to express Shh (Traiffort et al., 2001; Sanchez-Camacho and Bovolenta, 2008). However, there is no experimental evidence for either of these hypotheses. Moreover, an effect of Shh on patterning of the tectum or on the differentiation and maturation of the RGCs has not been taken into account. Additional experiments will be required to definitively demonstrate that Shh is directly required for axonal targeting in the tectum.

A patterning effect of Shh is not so far-fetched, as a recent study in zebrafish embryos suggests that indeed Shh guides RGC axons within the eye via an indirect mechanism, by patterning the optic stalk (Stacher Hörndli and Chien, 2012). The authors used transplantation studies to functionally test for the cell autonomy of Shh pathway components during intraretinal RGC axon guidance. When retinal precursor cells (RPCs) from wildtype donors were transplanted into hosts lacking *shh* or *smo*, RGC axons were misguided. Conversely, RPCs from donors lacking *shh* or *smo* projected normally when transplanted into wildtype hosts. These findings indicated that both Smo and Shh were acting non-cell-autonomously in intraretinal axon pathfinding, suggesting that Shh was required to indirectly pattern the eye rather than as a direct guidance cue.

To confirm this result, Stacher Hörndli and Chien (2012) applied pharmacological inhibitors of the Shh pathway during specific stages of embryonic development. While early application of the Smo antagonist SANT75 caused severe intraretinal pathfinding errors in most embryos, SANT75 treatment at the onset of RGC differentiation generated a significantly weaker phenotype, with most embryos displaying normal RGC pathfinding. Thus, Shh signaling was required during optic vesicle patterning, but not during RGC axon projection out of the eye. Indeed, the expression of several optic stalk markers was reduced or absent in *shh* and *smo* mutants, including Pax2, *netrin1, chemokine ligand 12a* (*cxcl12a*), and its homolog *cxcl12b*.

Unlike in mouse (Deiner et al., 1997), the loss of netrin1 by morpholino injection did not affect intraretinal RGC axon pathfinding in zebrafish. Rather, Stacher Hörndli and Chien (2012) examined whether the downregulation of chemokine signaling at the optic disc might be responsible for the Shh-induced RGC phenotype. Strikingly, *cxcl12a* mutants exhibited highly penetrant intraretinal RGC pathfinding errors, which resembled the phenotypes observed in Shh pathway mutants. Ectopic expression of Cxcl12a and additional transplantation experiments revealed that Cxcl12a was a direct chemoattractant for RGC axons. Additionally, the Shh and chemokine pathways interacted genetically to mediate RGC axon guidance inside the eye. Taken together, this study provided evidence that in zebrafish, Shh does not directly guide RGC axons toward the optic disc. Rather, Shh is required earlier in development to correctly pattern the eye and induce the expression of chemokines. In turn, Cxcl12a acts as an attractant for RGC axons inside the eye (Stacher Hörndli and Chien, 2012). This role in optic stalk patterning was consistent with a previous report that Shh signaling indirectly regulates axon pathfinding at the zebrafish midline by determining the expression of Slit guidance molecules, which in turn govern the positioning of midline glia (Barresi et al., 2005).

In contrast to Shh, where an effect of RGC axon targeting in the tectum has not yet been convincingly demonstrated, Wnts clearly influence topographic map formation in the visual system (Figure 3). In the chicken optic tectum, the morphogen Wnt3 is expressed in a medial^high^ to lateral^low^ gradient at the stage when RGC axons arrive (Schmitt et al., 2006). Wnt3 affects medio-lateral maps due to a biphasic effect. At low concentrations, Wnt3 inhibits the growth of ventral RGC axons, whereas at high concentrations, Wnt3 inhibits the growth of both dorsal and ventral RGC axons. The Wnt receptor Ryk, which is expressed in the RGCs in a ventral^high^ to dorsal^low^ gradient, mediates RGC axon growth inhibition. However, Wnt3 not only inhibits the growth of axons, it also stimulates the growth of dorsal RGC axons to the lateral tectum at low concentrations. In this case, the growth-promoting effect of Wnt3 is mediated by Fz receptors (Schmitt et al., 2006). All in all, Wnt3 is a lateral mapping force that counterbalances the medial mapping force exerted by EphrinB1-EphB signaling (Hindges et al., 2002).

### Descending nerve pathways

**Figure 4:**
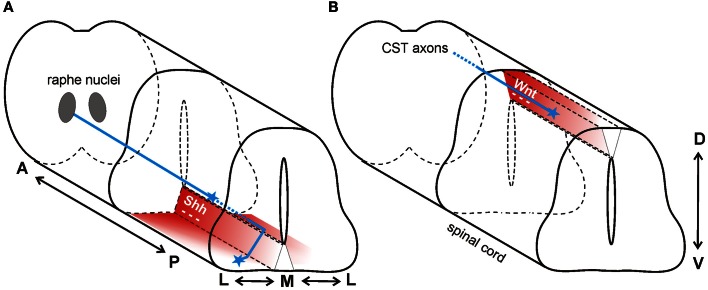
**Wnt and Shh are repellants for descending axon tracts of the brain. (A)** In mouse, Shh repels axons of the raphe spinal tract (blue) multidirectionally. In the anterior spinal cord, Shh (red) is expressed in an anterior^high^ to posterior^low^ (A-P) gradient, and in a medial^high^ to lateral^low^ (M-L) gradient. Thus, RST axons are pushed to grow both posteriorly and laterally. **(B)** In mouse, a decreasing anterior^high^ to posterior^low^ Wnt gradient (red) in the roofplate of the dorsal spinal cord repels descending corticospinal tract axons (blue) posteriorly.

Axons of projection neurons from the brain travel in descending tracts within the white matter of the spinal cord. Descending axons arise from various locations in the brain and synapse primarily on spinal interneurons, thus enabling the regulation of posture, movement, pain and sensation. Both Shh and Wnt are implicated in the repulsive guidance of descending nerve tracts (Figure 4).

Serotonergic axons from the caudal raphe nuclei (CRN) descend ipsilaterally and project to the caudal-most regions of the spinal cord, forming the raphe-spinal tract (RST). Along their trajectory, these serotonergic axons are specifically restricted to the ventral and ventrolateral funiculus. In contrast to what was observed in the posterior spinal cord in chick (Bourikas et al., 2005) and rat (Yam et al., 2012), Song et al. (2012) reported a decreasing anterior^high^ to posterior^low^ gradient of Shh in the mouse spinal cord at the cervical and thoracic levels. The authors hypothesized that floorplate-derived Shh diffusing away from its source would form gradients not only in the dorsoventral (D-V) and anteroposterior (A-P) axes, but also in the mediolateral (M-L) axis during guidance of serotonergic RST axons. These gradients of repulsive Shh would not only direct axons posteriorly down the spinal cord, but would also restrict the RST axons to the ventral and ventrolateral funiculus (Figure 4A).

Using conventional *in vitro* assays, Song and colleagues reported that Shh-expressing cells strongly repelled the outgrowth of axons emanating from CRN explants (Song et al., 2012). Extending these results, the authors developed an A-P graded guidance assay, in which CRN explants were placed at different positions along the A-P axis of dissected ventral spinal cords, which were expected to differentially secrete diffusible factors according to their A-P expression patterns. In this assay, CRN axons were consistently repelled at all levels along the A-P axis, but the effects were stronger when the CRN explants were cultured in proximity to the anterior spinal cord. This finding was consistent with a diffusible axon guidance cue from the ventral spinal cord establishing a high-to-low repulsive gradient that would direct RST axons toward the caudal spinal cord. Shh was demonstrated to act in such a manner, since function-blocking antibodies restored the growth of CRN axons in these co-cultures. Thus, serotonergic RST axons, like dI1 commissural axons of the lumbar spinal cord (Bourikas et al., 2005), are guided along the longitudinal axis by a repulsive gradient of Shh.

Next, Song et al. (2012) asked whether a gradient of repulsive Shh in the mediolateral axis of the ventral spinal cord could act similarly, thereby restricting RST axons to the ventral and ventro-lateral funiculus. To test this idea, CRN explants were co-cultured next to neural tube slices taken from different medial to lateral locations. Again, axons displayed differential responsiveness in this *in vitro* assay, with medial neural tube slices suppressing outgrowth more strongly than lateral slices. This graded response was abolished by the addition of Shh pathway blockers.

*In vivo* experiments supported the findings of the *in vitro* assays. Song et al. (2012) examined RST axon pathfinding in embryos in which Shh signaling was perturbed by different means by analyzing *Shh* hypomorphic mice, mice with conditional inactivation of *Smo*, and mice that were electroporated *in utero* with a Shh-insensitive version of Ptc. In all case, neural tube patterning was unaffected, but the formation of the RST was abnormal. The phenotypes in the three conditions were not identical, however. While interference with Ptc and Smo led to stalling of the RST axons, a failure to innervate the posterior spinal cord and abnormal invasion of the medial neural tube, the *Shh* hypomorphs displayed a reduced number of axons extending into the spinal cord. Over-activation of the Shh pathway (by the expression of a constitutively active version of Smo) caused RST axons to loop back and deflect to the brainstem, suggesting that increased Shh-Smo repulsion pushed the descending axons away from the cervical spinal cord. Taken together, these findings showed that Shh guides serotonergic RST axons multidirectionally. Shh not only directs the posterior growth of these axons in the longitudinal axis, but is also required to position the descending tracts in their appropriate mediolateral position in the ventral spinal cord. In addition to identifying a new Shh-responsive population of axons, this study revealed a novel, efficient mechanism of axon guidance along several axes: simultaneous, multidirectional guidance by a single molecule.

In addition to the Shh gradient mentioned above for the guidance of serotonergic axons of the raphe nucleus, there is also an anterior^high^ to posterior^low^ expression gradient of *Wnt1* and *Wnt5a* at cervical and thoracic levels of the mouse spinal cord (Liu et al., 2005). In contrast to the attractive effect of Wnt4 in the ventral neural tube, Wnt1 and Wnt5a had a repulsive effect on descending corticospinal tract axons (Figure 4B). Axons extending from cortical explants derived from postnatal day 0 (P0) brains were repelled by Wnt1 and Wnt5a in a Ryk-dependent manner (Liu et al., 2005). *In vivo* perturbation of Ryk by injection of function-blocking antibodies into the spinal cord produced abnormal guidance of CST axons, consistent with its role as a mediator of a repulsive cue *in vitro* (Liu et al., 2005).

### Dopaminergic neurons of the midbrain and brainstem

Midbrain dopaminergic neurons (mDNs) are a functionally diverse population, which is reflected by a structural heterogeneity in their axonal projections. In mammals, mDN axons project rostrally toward the forebrain, where they expand both dorsoventrally and mediolaterally. The axons from the medially located ventral tegmental area (VTA) project along a ventromedial course and primarily target more medial target tissues in the forebrain, while the more lateral axons originating from the substantia nigra (SN) project dorsolaterally and target lateral forebrain regions. Several classical guidance cues have been implicated in mDN pathfinding, including Netrins (Lin et al., 2005), Slits (Bagri et al., 2002), Ephrins (Sieber et al., 2004), and Semaphorins (Yamauchi et al., 2009). In addition, Shh was recently identified as a chemoattractant for mDN axons both *in vitro* and *in vivo* (Hammond et al., 2009). On the other hand, Wnts may either attract or repel mDN axons (Fenstermaker et al., 2010).

Shh is expressed in the ventral midline (floorplate) of the midbrain during the time that mDN axons traverse rostrally (E12.5-E15.5 in mouse). As in other regions along the neural tube, Shh forms a ventral to dorsal gradient within this area (Ericson et al., 1995). mDN axons express Ptc and Smo, but interestingly, they do not express other components of the canonical Shh pathway, such as Gli1, Gli2, or Gli3, during this time (Hammond et al., 2009). Explants containing mDN neurons projected axons toward a source of Shh, an effect that could be blocked by addition of the Smo antagonist, cyclopamine. These results suggested that Shh was an attractive cue for mDN axons. Consistent with this idea, Smo was also required for mDN axon guidance in the intact CNS. Hammond et al. (2009) took advantage of conditional mutant mice (*Nestin-Smo* ko mice) in which Smo is specifically and completely inactivated in the central nervous system by E11.5. In these embryos, the patterning and specification of the embryonic midbrain (including mDNs) and their forebrain targets are unaltered, since canonical Shh signaling is no longer required for these processes after E10 (Hynes et al., 1995; Blaess et al., 2006). Thus, the requirement for Shh signaling during the guidance of mDN axons could be assessed independently of its earlier roles in tissue patterning. In the conditional Smo knockout mouse, the lateral mDN projected axons normally toward their rostral targets, whereas projections from the medial mDN were misdirected. Within this abnormal population, the ventral-most fibers were more severely affected, suggesting a role for Shh signaling specifically in the ventral targeting of medial dopaminergic axons. In support of this, co-culture assays showed that medial, but not lateral, mDN axons were attracted to a Shh source. An open question is how this difference in sensitivity to Shh is achieved at the molecular level, since both populations of mDNs express Smo at similar levels. Therefore, another unidentified modulator of Shh signaling must be differentially expressed in these cell populations.

In the midbrain, Wnt5a is expressed in an anterior^low^ to posterior^high^ gradient whereas Wnt7b is expressed in an anterior^high^ to posterior^low^ gradient. *In vivo, Wnt5a*^−/−^ mice exhibit only minor and transient posterior projections of mDN axons. *In vitro*, axons of cultured mDN neurons are repelled by exogenous Wnt5a, whereas these axons are attracted by exogenous Wnt7b (Fenstermaker et al., 2010). These effects of Wnt ligands on mDN axons were abolished when open-book explants from *Frizzled3*^−/−^ mice were co-cultured *in vitro* with a Wnt source (Fenstermaker et al., 2010). Therefore, Wnt5a repels mDN axons and Wnt7b attracts them, in a Fz3-dependent fashion (see also Section Molecular mechanisms of Wnt-mediated axon guidance). The mild and transient effects observed in the *Wnt5a*^−/−^ mice might therefore be explained by the combinatorial, collaborative effects of Wnt5a and Wnt7b during normal development. The remaining attractive Wnt7b activity may be sufficient to overcome loss of Wnt5a *in vivo*.

## Molecular mechanisms of axon guidance mediated by Shh

### Chemoattraction

An intracellular signaling cascade that mediates the chemoattractive guidance response to Shh has been described in rodent commissural neurons (Yam et al., 2009). Previous reports indicated that commissural axons express Smo (Charron et al., 2003; Yam et al., 2009) and Boc (Okada et al., 2006), and that these proteins are required for the attraction of axons toward an increasing gradient of Shh derived from the floorplate (see Section dI1 commissural axons of the spinal cord). The possible contribution of Ptc to the chemoattractive effect of Shh is yet to be studied.

Shh signaling could occur via at least two pathways: (1) Boc/Smo could elicit a canonical Shh signal in the nucleus via Gli-dependent transcriptional changes, or (2) Shh could act locally at the growth cone through an alternative, transcription-independent pathway. In order to distinguish between these possibilities, Yam et al. (2009) used an *in vitro* axon guidance assay for commissural neurons. In a so-called Dunn chamber, dissociated commissural neurons growing on coverslips were exposed to stable gradients of axon guidance molecules. Axon responses to these gradients could be assayed within 1–2 h, thus enabling short-term pathways (i.e., those likely to be transcription-independent) to be identified and analyzed.

Dissociated commissural axons responded very swiftly to a gradient of Shh in this assay. The addition of transcriptional inhibitors had no effect, supporting the idea that the chemoattractive turning response to Shh occurred independently of transcription. Accordingly, the expression of Gli3R, a dominant repressor of Gli-mediated transcription (Persson et al., 2002), did not affect the ability of commissural axons in explants to turn toward a source of Shh.

Further experiments revealed that Shh attracts commissural axons by activating Src family kinases (SFKs) in the growth cone, in a Smo- and Boc-dependent manner (Yam et al., 2009). In the presence of a Shh gradient, the SFKs Src and Fyn were rapidly phosphorylated and asymmetrically distributed in the growth cone of commissural neurons. This local, polarized response elicited growth cone turning, since SFKs can modulate cytoskeletal rearrangement and filopodial dynamics (Suter and Forscher, 2001; Robles et al., 2005; Liu et al., 2007). Thus, in attractive commissural axon guidance, Shh signals via a rapid, local, transcription-independent mechanism (Figure 2A).

It is currently unknown whether the same pathway is activated in RGC neurons and/or dopaminergic neurons to elicit chemoattraction toward Shh. However, in ipsilateral RGC axons, Boc has been described as the receptor responsible for the repulsion of axons from Shh at the chiasm (Fabre et al., 2010; see Section Retinal ganglion cell axons and topographic mapping), rather than as an attractive guidance receptor. The expression and role of Boc in dopaminergic neurons has not been investigated to date.

### Chemorepulsion

In commissural axon guidance, the axonal response to floorplate-derived Shh switches from attraction in the dorsoventral axis to repulsion in the longitudinal axis, within just a few hours. In the chick, this switch in responsiveness is due to a change in the expression of Shh receptors on the axons. Pre-crossing commissural axons which express Boc and Smo are attracted to Shh via a non-canonical SFK-mediated pathway (Okada et al., 2006; Yam et al., 2009). Post-crossing axons express Hhip and are repelled by Shh (Bourikas et al., 2005). However, the intracellular signaling mechanisms transduced by Hhip are currently unknown. Hhip is a type-I transmembrane protein (Chuang and McMahon, 1999), but its short cytoplasmic tail seems unlikely to directly induce intracellular signals which influence growth cone turning. Rather, Hhip could be involved as a Shh-binding unit in a co-receptor complex with an unidentified signaling receptor (Figure 2B).

In mouse, a recent report suggests that the switch in responsiveness to Shh that occurs at the midline is due to a cell-intrinsic timer mechanism, mediated by 14-3-3 proteins (Yam et al., 2012). Using again their Dunn chamber assay, Yam et al. (2012) confirmed the direct repulsive effect of Shh on post-crossing commissural axon turning *in vitro*, as demonstrated earlier (Bourikas et al., 2005). Interestingly, the response of the axons to a Shh gradient changed according to the number of days they had been in culture. While commissural axons at 2 days *in vitro* (DIV) were attracted up a Shh gradient (Yam et al., 2009, 2012), neurons at 3-4 DIV switched their direction of growth, turning away from higher concentrations of Shh. Based on these findings, the authors suggested that the response of commissural neurons to Shh might change over time, via a cell-intrinsic mechanism even in the absence of floorplate contact.

Previous work had implicated 14-3-3 adaptor proteins in repulsive neuronal responses, and they had been localized to growth cones (Kent et al., 2010). Both 14-3-3β and 14-3-3γ were enriched in post-crossing commissural axons, consistent with their ability to mediate anteroposterior guidance responses (Yam et al., 2012). Furthermore, these proteins increased in expression over time *in vitro*, consistent with their potential involvement in the time-dependent change in Shh responsiveness. To test this hypothesis, 14-3-3 proteins were antagonized *in vitro* with pharmacological inhibitors or by electroporation of a plasmid encoding a shRNA construct, and the response of 3DIV commissural axons to a Shh gradient was assayed. A reduction in 14-3-3 activity shifted the repulsive response of 3DIV commissural neurons from repulsion to attraction. *In vivo*, inhibition of 14-3-3 prevented the correct anterior turn in a subset (<35%) of post-crossing commissural axons, an effect that was dependent on PKA activity. Results from the converse gain-of-function experiments were consistent with the idea that 14-3-3 proteins can regulate a cell-intrinsic, temporal change in turning responses to Shh (Yam et al., 2012). However, there are several outstanding questions arising from this study. For example, it remains to be shown how a gradual upregulation of 14-3-3 proteins can be causally linked to an abrupt switch in responsiveness of the growth cone in the floorplate. It is also unknown how a 14-3-3-dependent change in PKA activity regulates the growth cone responsiveness to Shh, since the levels of cAMP were unchanged in 4DIV growth cones compared to 2DIV growth cones (Yam et al., 2012). Additionally, the growth cone receptor responsible for detecting the longitudinal gradient of Shh in post-crossing axons remains to be identified, although Smo is apparently involved. Finally, although the authors claim that the commissural neurons used in the Dunn chamber assays are floorplate naïve, they were isolated from E13 rats. At this age (equivalent to E11.5 in mouse or HH23-24 in chick), many commissural axons have clearly reached the ventral midline and some have even crossed the floorplate and turned into the longitudinal axis (Bovolenta and Dodd, 1990; Ruiz de Almodovar et al., 2011). Therefore, it is possible that many of the isolated neurons used in these experiments had already been under the influence of a floorplate-derived cue.

**Figure 5:**
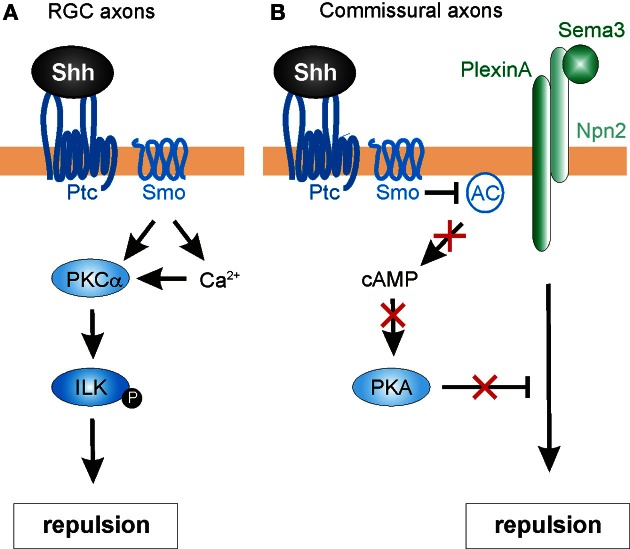
**Intracellular mechanisms mediating Shh-induced repulsive axon guidance. (A)** In RGC axons, Shh binding to Ptc promotes the Smo-dependent activation of protein kinase Cα (PKCα), which in turn phosphorylates integrin-linked kinase (ILK). ILK promotes repulsive axon turning. **(B)** In commissural neurons, Shh acts through Ptc and Smo to block adenylyl cyclase (AC) activity. This lowers cAMP levels and inhibits protein kinase A (PKA) activity. In turn, this confers growth cone sensitivity to class 3 semaphorins (Sema3), by allowing repulsive signaling downstream of a PlexinA-Npn2 complex.

Another pathway mediating the negative guidance effects of Shh has recently been described in chick RGC axons (Guo et al., 2012). Here, the authors investigated the involvement of protein kinase C (PKC) family members in Shh-mediated repulsive axon guidance (Figure 5A). The PKC family of serine/threonine kinases is comprised of several members that are sub-categorized as conventional (α, β I, β II, γ), novel (δ, ε, η, θ), or atypical (ζ, λ), based on their second-messenger requirements (Steinberg, 2008). Conventional PKCs require diacylglycerols (DAG) and Ca^2+^ for their activation, while novel PKCs require DAG only and atypical PKCs require neither. PKCs were previously implicated in canonical Shh signaling (Riobo et al., 2006) and in repulsive axon guidance (Kolpak et al., 2009), but the specific activities of different family members and their substrates was not well understood. Using pharmacological inhibitors in RGC cultures, Guo et al. (2012) showed that PKCα played a specific role in RGC growth cone collapse in response to Shh. Additionally, Shh rapidly activated PKCα, and led to a Smo-dependent increase in Ca^2+^ in the growth cone.

Next, the authors sought a downstream target of PKCα that might mediate the repulsive guidance effects of Shh. One predicted cytoplasmic target was integrin-linked kinase (ILK), an important scaffolding protein that can link cell adhesion and growth factor signaling to the actin cytoskeleton (Hannigan et al., 2005) and had previously been implicated in neurite outgrowth (Ishii et al., 2001) and neuronal polarity determination (Guo et al., 2007). Indeed, Shh stimulation significantly increased the levels of phosphorylated ILK in RGC axons, an effect that was inhibited by pharmacological blockade of PKCα. Disruption of PKCα or ILK by the expression of mutant constructs or pharmacological inhibitors also caused a significant reduction in both Shh-induced macropinocytosis (a clathrin-independent endocytosis pathway mediating growth cone collapse; Kolpak et al., 2009) and repulsive axon turning (Guo et al., 2012). Taken together, the results indicated that that PKCα and ILK were required for the negative axon guidance effects of Shh.

Finally, Guo et al. (2012) assessed the roles of PKCα and ILK in Shh-mediated RGC axon guidance *in vivo*. The optic vesicles of chicken embryos were injected with RCAS viruses expressing GFP alone, or GFP fused with dominant-negative PKCα, or ILK-DM (a double-mutant ILK construct that could not be phosphorylated by PKCα). Indeed, the expression of DN-PKCα or ILK-DM resulted in misprojection of RGC axons into the ipsilateral optic tract and contralateral optic nerve at the optic chiasm, consistent with a disruption in Shh-mediated signaling at the chiasm. Interestingly, neither DN-PKCα nor ILK-DM affected the projection of RGC axons within the retina toward the optic disc (see Section Retinal ganglion cell axons and topographic mapping). This finding was consistent with PKCα and ILK specifically mediating the negative guidance effects of high concentrations of Shh on RGC axons. ILK-DM was less effective than DN-PKCα in inhibiting Shh-induced repulsive effects. This observation suggests that ILK is not the sole effector of PKCα signaling. The identification of other cytoplasmic targets is open to future research.

### Indirect regulation of axon guidance by modulation of cyclic nucleotides

In addition to acting directly as an axon guidance cue, Shh may also influence growth cone responsiveness via local intracellular mechanisms. One cytoplasmic, non-canonical target of Shh signaling is the cyclic nucleotides, which can modulate signaling responses to axon guidance cues (Song et al., 1997). The ratio of cAMP/cGMP determines attractive or repulsive axonal responses (Song et al., 1998), with attraction being favored by cAMP^high^/cGMP^low^, and cAMP^low^/cGMP^high^ favoring repulsion. Since Shh can reduce the activity of protein kinase A (PKA), it can therefore influence cyclic nucleotide levels, thus modulating the responsiveness of axons to guidance cues.

This hypothesis is supported from several studies. For example, chick RGC axons not only display a lack of outgrowth following exposure to Shh, but also exhibit a marked reduction in cytoplasmic cAMP (Trousse et al., 2001). More directly, a recent study indicates that Shh modulates cAMP levels in commissural axons as they reach the floorplate (Parra and Zou, 2010) (Figure 5B). This acts as a molecular “switch,” allowing commissural axons to acquire responsiveness to midline repellants and escape to the contralateral side. The authors found that disruption of Shh signaling (via shRNA-mediated knock down of Smo, function-blocking antibodies or the expression of a dominant-negative form of Ptc) resulted in severe guidance defects in commissural axons. The abnormalities were assessed in open-book preparations, revealing phenotypes that included stalling/knotting in the floorplate, randomized turning, and overshooting of post-crossing axons, and recrossing of the midline. These abnormalities resembled those found in embryos deficient for Neuropilin-2 (Npn2), a receptor component for Sema3B and Sema3F, two secreted class-3 Semaphorins (Zou et al., 2000). Sema3B/3F provide important chemorepulsive signals that, together with the Slits, expel commissural axons out of the midline. Commissural axons only respond to these repellants after reaching the floorplate, suggesting that a floorplate-derived signal, such as Shh, might mediate a switch in guidance responses. Indeed, Parra and Zou (2010) found that pre-crossing commissural axons were only repelled by Sema3B/3F *in vitro* after exposure to Shh. Cyclic nucleotides were shown to be important for this phenomenon. The authors first examined whether cAMP/PKA activity was needed for proper midline axon pathfinding. Indeed, increasing the level of cAMP in the explant cultures by application of the adenylyl cyclase activator forskolin caused similar defects on commissural axons as seen with disrupted Shh signaling. Additionally, forskolin attenuated the Shh-induced repulsive response to Sema3B/3F by pre-crossing commissural axons. Together, these findings suggest that Shh regulates cAMP levels, which in turn modulates the sensitivity of growth cones to Semaphorins (Figure 5B). In agreement with this model, PKA activity has previously been shown to be coupled with Semaphorin-PlexinA-mediated repulsion *in vivo* (Terman and Kolodkin, 2004). However, the precise mechanism by which the Shh-mediated cAMP level activates Sema3 signaling in vertebrates is unknown. Taken together, these findings identify Shh not only as a direct chemotropic cue, but also as a midline-derived switch that activates growth cone responses to other axon guidance molecules.

### Canonical Shh signaling in axon guidance?

**Figure 6:**
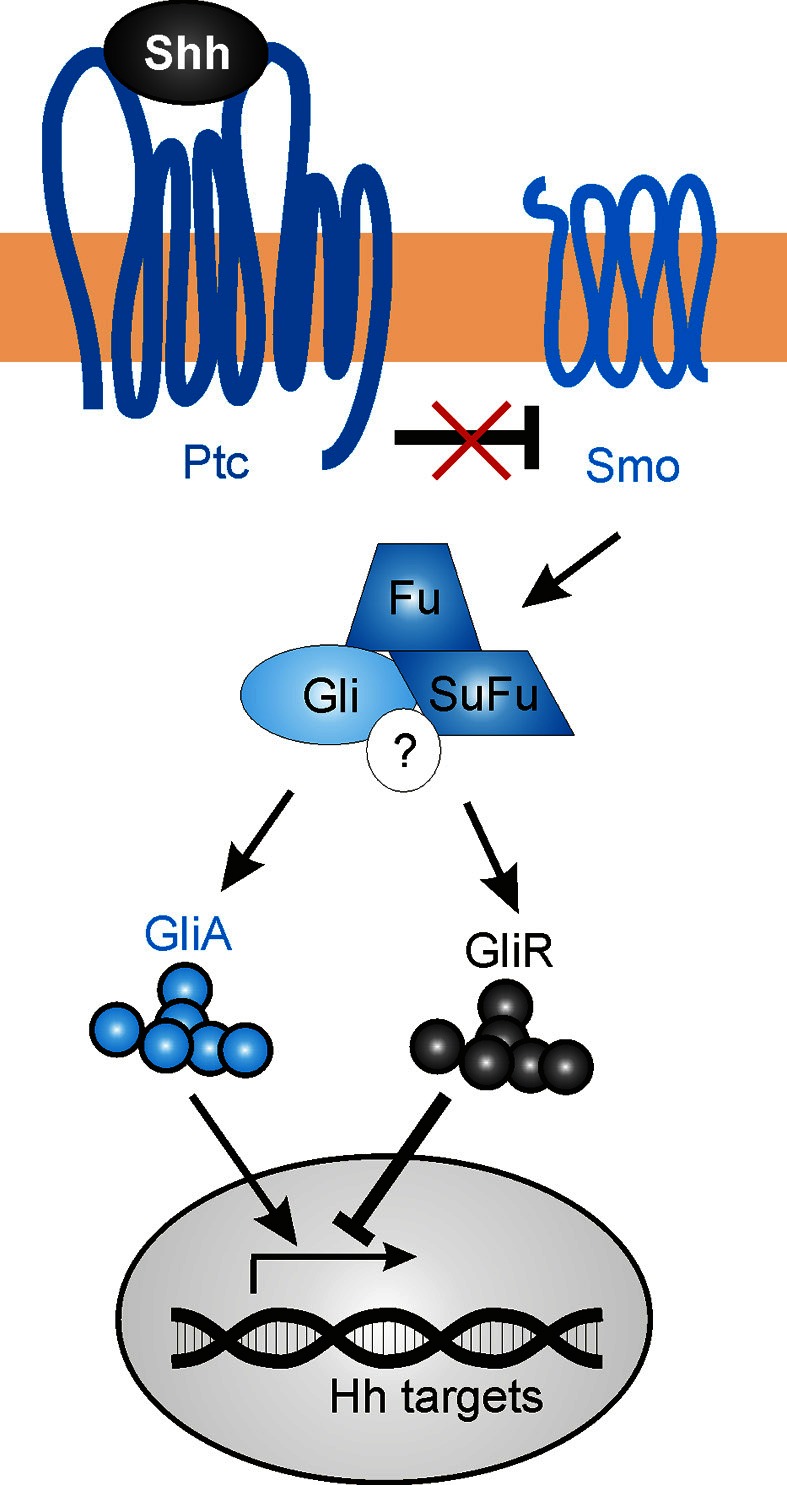
**The canonical Shh signaling pathway**. In the absence of Shh, Gli transcription factors are proteolytically processed to repressor forms (GliR), which block the transcription of target genes. Shh binding to the 12-pass transmembrane receptor Patched (Ptc) relieves the inhibition of Smoothened (Smo), which in turn promotes the accumulation of the activator forms of Gli (GliA) while suppressing GliR production. The regulation of Gli activity occurs via an intracellular complex composed of Fused (Fu), Suppressor of Fused (SuFu) and possibly other components (?).

The mechanisms described above delineate several non-canonical mechanisms by which Shh guides axons or modulates growth cone responsiveness. However, Shh's transcriptional activity might also contribute to correct axon pathfinding, by regulating the levels and types of guidance receptors and/or modulators of signaling that are expressed (Figure 6). The transcriptional activity of Shh has been shown to indirectly regulate axon guidance both in the spinal cord (Domanitskaya et al., 2010; see Section dI1 commissural axons of the spinal cord) and in the retina (Stacher Hörndli and Chien, 2012; see Section Retinal ganglion cell axons and topographic mapping) via the cell-non-autonomous induction of cues (Sfrps and chemokines) that indirectly or directly mediate growth cone behavior. However, the role of Shh canonical signaling in the navigating neurons themselves has not been extensively studied to date, especially in an *in vivo* context in which multiple guidance cues are presented simultaneously.

In a speculative view, navigating axons that approach a Shh source could activate both a non-canonical guidance response, as well as initiating transcriptional activity in the nucleus. As the axons encounter increasing levels of Shh expression, there is a progressive upregulation of Shh-induced genes. Some of these directly affect the signaling response to Shh, including Ptc and Hhip, while others could activate other signaling pathways that mediate axon guidance (such as EphB4, EphrinB2, PlexinA2, or Adamts1) (Chuang and McMahon, 1999; Oliver et al., 2003; Yu et al., 2008). In sum, Shh-induced transcriptional activity could alter the growth cone sensitivity to numerous guidance factors as axons navigate toward their targets, allowing the axons to respond dynamically to changing environments.

## Molecular mechanisms of Wnt-mediated axon guidance

**Figure 7:**
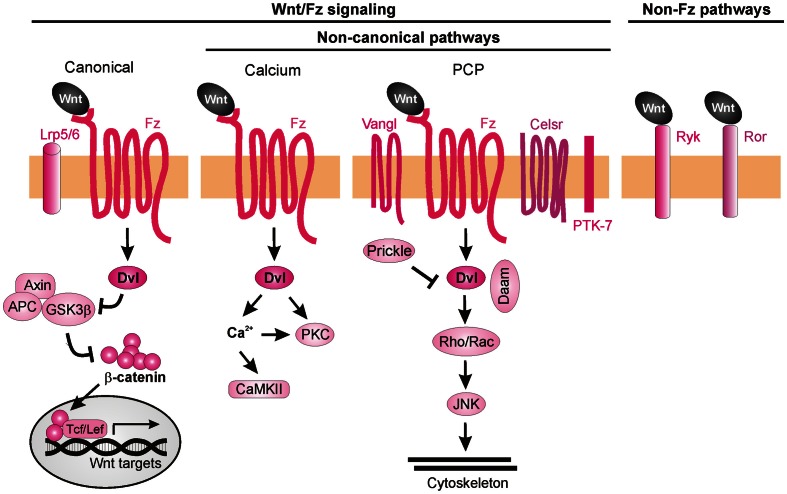
**Wnt signaling pathways**. Wnt ligands can transduce their signal through at least three Frizzled-dependent (Fz) pathways: the canonical, the calcium, and the PCP pathways, all of which involve the activation of Dishevelled (Dvl). The canonical pathway requires the co-receptors Lrp5/6 to recruit Dvl and inhibit the “destruction complex” composed of Adenomatous polyposis coli (APC), Axin, and Glycogen synthase 3β (GSK3β). Wnt binding leads to the phosphorylation of GSK3β in this complex and, as a consequence, to the accumulation of unphosphorylated β-catenin, which can enter the nucleus and together with the transcription factors Tcf/Lef induce the expression of target genes. Activation of the calcium pathway results in an increase of cytosolic calcium (Ca^2+^) and the subsequent activation of calcium-dependent kinases (CaMKII). In the PCP pathway, activation of the transmembrane proteins Vangl, Celsr, and PTK7 and the recruitment of the intracellular proteins Prickle and Daam lead to activation of Rho GTPases and JNK, promoting cytoskeleton remodeling. More recently, Fz-independent Wnt signaling has been described. Wnt ligands can bind directly to receptors such as Ryk or Ror. The intracellular signaling cascades activated in these cases are poorly understood.

Wnt ligands are able to activate a variety of receptors on the cell surface (van Amerongen et al., 2008). Initially, Wnt signaling was subdivided into three pathways, the canonical (also known as β-catenin-dependent) pathway, the PCP pathway, and the Wnt/Ca^2+^ pathway (Figure 7). Considering the many Wnt functions during post-morphogenesis Wnt signaling has become a complex network of interactors (van Amerongen and Nusse, 2009; Clark et al., 2012; Nusse, 2012; Salinas, 2012). Upon binding of the Wnt ligands to Fz, the intracellular protein Dishevelled (Dvl) is activated and can initiate all three signaling cascades: the canonical (β-catenin-dependent) pathway, the calcium (Ca^2+^) pathway and the PCP pathway. In the canonical Wnt pathway, stimulation of Dvl leads to inactivation of GSK3β, preventing it from phosphorylating β-catenin. Unphosphorylated β-catenin is no longer degraded by the proteasome and is able to enter the nucleus, where it interacts with the Tcf/Lef transcription factors to activate the transcription of target genes (Macdonald et al., 2007; Nusse, 2012). In the Wnt/Ca^2+^ pathway, Wnt ligands induce an increase in intracellular calcium leading to the activation of PKC and calcium/calmodulin-dependent protein kinase II (CaMKII) (Kohn and Moon, 2005; Semenov et al., 2007). The PCP pathway mediates tissue polarity and is less understood than canonical Wnt signaling in terms of biochemical interactions. It involves the participation of transmembrane proteins such as Flamingo (a.k.a. Celsr), Van Gogh-like proteins (Vangl, a.k.a Strabismus) and Protein tyrosine kinase 7 (PTK7), as well as intracellular proteins such as Prickle, Diego, and Daam. The activation of the PCP pathway leads to stimulation of Rho and Rac GTPases and the consequent rearrangement of the cytoskeleton (Semenov et al., 2007; Wang and Nathans, 2007; Simons and Mlodzik, 2008).

Even though several Wnt ligands and their axon guidance receptors have been identified, little is known about the intracellular cascades that lead the growth cones to respond to a certain cue. Despite the participation of canonical Wnt signaling in *C. elegans* anteroposterior axon guidance (Maro et al., 2009), there is so far no evidence for the involvement of this pathway in vertebrate axon guidance. In fact, mice mutant for Lrp6 (an indispensable co-receptor in the canonical pathway) do not exhibit major pathfinding defects (Lyuksyutova et al., 2003), suggesting that the axon guidance functions of Wnt are not mediated by canonical signaling events. The non-canonical Wnt signaling pathways that have been implicated in axon guidance are described below.

### PKC-PI3K signaling in commissural axon guidance

**Figure 8:**
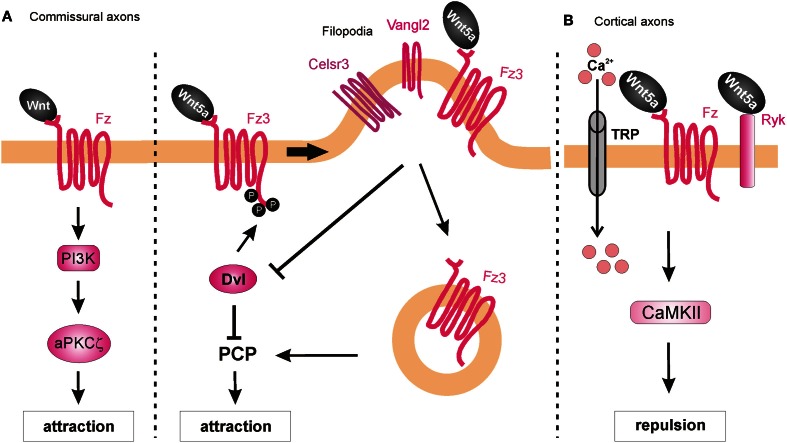
**Intracellular mechanisms underlying Wnt-induced axon guidance. (A)** Wnts promote commissural axon attraction via the activation of the PI3K-atypical PKCζ signaling and the PCP pathway. The PCP pathway is inhibited through the Dvl-dependent phosphorylation of Fz3, which as a result remains in the plasma membrane. The local expression of Vangl2 in the filopodia of the growth cone prevents the Dvl-induced phosphorylation of Fz3 upon binding of Wnt5a and results in the internalization of Fzd3 and the activation of the PCP pathway. **(B)** Wnt5a repels cortical axons by binding to both Fz and Ryk receptors, which allow the entrance of calcium via the TRP channels. Activation of Ryk alone results in axon growth promotion, whereas co-activation of Ryk and Fz is required for axon repulsion.

Searching for a role of PKC in post-crossing commissural axon guidance, Wolf et al. (2008) found that a calcium-independent PKC pathway is involved. The general pharmacological inhibition of all PKCs resulted in abnormal turning of post-crossing commissural axons in open-book explants, whereas inhibition of only conventional PKCs did not affect A-P turning (Wolf et al., 2008). Taken together, these data suggest that non-conventional PKC signaling (i.e., calcium-independent) is involved in axon turning. Pan-inhibition of PKCs also perturbed Wnt4-induced axon outgrowth. Treatment of the explants with a specific inhibitor of the atypical PKCζ showed similar guidance defects. The pharmacological studies were confirmed with the expression of a dominant-negative form of PKCζ in rat spinal cords, where a significant increase of post-crossing commissural axons that turned caudally was seen (Wolf et al., 2008). Since PKCζ can be activated by phosphatidylinositol-3-kinase (PI3K) signaling, the authors pharmacologically blocked this pathway using the PI3K inhibitor wortmannin, and found randomization of turning by post-crossing commissural axons. Furthermore, inhibition of PI3Ks by expression of a kinase-defective form of PI3Kγ in the spinal cord caused aberrant phenotypes of post-crossing commissural axons. Interestingly, axons that expressed the wild-type form of the PI3Kγ kinase domain (p110-WTγ) were observed to turn into the longitudinal axis before crossing the floorplate. In addition, overexpression of p110-WTγ in pre-crossing commissural neurons, which are normally not responsive to Wnt4, became sensitive to Wnt4 attraction (Wolf et al., 2008). The effect of p110-WTγ in explants and cultured cells suggests that PI3Kγ acts as a regulator that turns on the responsiveness of commissural axons to Wnt4 after midline crossing (Figure 8A).

### Ryk-dependent Wnt/Ca^2+^ signaling

Besides their role as attractive cues in post-crossing commissural axon guidance, Wnt ligands have also been shown to act as repulsive cues for post-crossing cortical axons that cross the midline in the corpus callosum (Keeble et al., 2006). In *Ryk*^−/−^ mutant mice, callosal axons are able to cross the midline but then fail to project away from the midline area, and thus, form aberrant axonal trajectories on the contralateral side. Keeble et al. (2006) found that *Wnt5a* is expressed around the corpus callosum at the time when cortical axons cross the midline. *In vitro* studies demonstrated that axons from cortical explants acquired sensitivity to Wnt5a at E18, concurrent with midline crossing. Responsiveness to Wnt5a was mediated by Ryk. However, *Ryk* mRNA levels were highest before midline crossing, thus it remains to be shown how Ryk-mediated repulsive signals are restricted to post-crossing callosal axons.

Further studies have shed more light on the roles of Ryk in mediating the responses of cortical axons to Wnt5a. Li et al. (2009) found that the graded application of Wnt5a to hamster cortical explants not only repelled cortical axons but also increased their rates of outgrowth. Thus, it appeared that Wnt5a could simultaneously activate two different processes in cortical axons: repulsion and outgrowth. These distinct responses were mediated by two different receptor compositions. While axonal outgrowth was carried out via Ryk alone, repulsion required the activities of both Ryk and Fz receptors. Intriguingly, the authors found that both the outgrowth and repulsive responses were dependent on calcium signaling, as cortical axons no longer showed any response to a Wnt5a gradient after chelation of cytoplasmic calcium. However, the specific pharmacological inhibition of IP3 receptors prevented outgrowth but not repulsive turning of cortical neurons in response to Wnt5a, while the blockade of TRP channels inhibited both Wnt5a-induced outgrowth and repulsion (Li et al., 2009). Thus, Wnt5a-induced axon outgrowth requires cytosolic calcium from both intracellular and extracellular sources, while Wnt5a-induced repulsion requires calcium only from extracellular sources. Next, Li et al. (2009) performed pharmacological inhibition of other calcium signaling components that were known for their role in axonal outgrowth. Inhibition of phospholipase C (PLC), which is upstream of IP3, or CaMKII, which is downstream of calcium signaling, prevented Wnt5a-induced outgrowth, thus confirming the role of Wnt/calcium pathway in axon outgrowth. The activity of Wnt/calcium pathway in the growth and guidance of dissociated cortical axons was further confirmed in a cortical slice model of the developing corpus callosum (Hutchins et al., 2011) (Figure 8B). Importantly, these studies showed that Fz receptors are able to mediate repulsion in vertebrate axon guidance, consistent with previous reports in *C. elegans* (Pan et al., 2006). Furthermore, the results showed that Ryk can promote axonal outgrowth, in addition to repulsion, in response to Wnt5a. However, it is still unknown how the growth cone can distinguish between the different calcium sources to mediate the distinct outputs.

### Wnt/PCP pathway in axon guidance

Recently, it was shown that Ryk can also regulate the PCP pathway by interacting with Vangl2 during vertebrate development (Andre et al., 2012; Macheda et al., 2012). Interestingly, Wnt5a enhances the biochemical interaction between Ryk and Vangl2, and regulates the stability of Vangl2 (Andre et al., 2012). Based on these findings, it would be interesting to determine whether Ryk signals through the PCP pathway to fulfill any of its roles in axon guidance. However, to date, there is no evidence supporting such a mechanism.

On the other hand, a role for the core PCP component Vangl2 in axon guidance has been demonstrated. Vangl2, Fz3, and Celsr3 are expressed in serotonergic and dopaminergic neurons in the midbrain at the time when these neurons extend their axons through the brainstem (Fenstermaker et al., 2010). The PCP pathway is involved in the guidance of serotonergic axons, since mice deficient in Frizzled3, Vangl2 (Loop-tail, Lp), and Celsr3 all show abnormal projections of both ascending and descending serotonergic axons. These mice also show posterior misprojections of dopaminergic axons, which normally project anteriorly. Wnt5a is expressed in local gradients in the brainstem: in the hindbrain, it is expressed in an anterior^high^ to posterior^low^ fashion, but switches to an increasing posterior gradient at the level of the rhombomere 4; while in the midbrain, Wnt5a shows an anterior^low^ to posterior^high^ gradient that is changed at the isthmus. In contrast, Wnt7b is expressed in an anterior^high^ to posterior^low^ gradient in the midbrain. Using open-book explants of the hindbrain from Fz3 mutant mice, Fenstermaker et al. (2010) found that serotonergic ascending axons were misprojected in response to Wnt5a, whereas dopaminergic axons showed a decreased repulsive response to Wnt5a and a decreased attractive response to Wnt7b (Fenstermaker et al., 2010). Therefore, Wnt5a-stimulated attraction of serotonergic neurons and repulsion of dopaminergic neurons, as well as Wnt7b-stimulated attraction of dopaminergic neurons is mediated by Fz3, which is known to act in the PCP pathway.

PCP signaling is also involved in A-P guidance of post-crossing commissural axons in the mouse. Celsr3, Fz3, and Vangl2 are all expressed in mouse commissural axons when they have to turn into the A-P axis (Torban et al., 2007; Shafer et al., 2011). Similarly, the phosphorylated version of JNK, which is a downstream component and represents a readout of PCP signaling activation (Boutros et al., 1998), is enriched in post-crossing commissural axons. Both the Lp mouse (which contains a destabilizing point mutation in the *Vangl2* gene) and the Celsr3 knockout mouse exhibit strong impairments of the directionality of post-crossing axons (Shafer et al., 2011), phenocopying the defects of the Fz3-deficient mouse (Lyuksyutova et al., 2003). Inhibition of JNK in open-book explant cultures also resulted in anteroposterior guidance defects. These results show that PCP signaling is responsible, at least in part, for the post-crossing commissural axon guidance *in vivo*. Shafer et al. (2011) also reported that in a heterologous system (HEK cells), co-expression of Dishevelled1 (Dvl1) with Fz3 reduced phospho-Jun levels after Wnt5a application compared to Fz3 overexpression alone, indicating that Dvl1 induced a feedback inhibition of the PCP pathway. This effect of Dvl1 was abolished by co-transfection of Vangl2, suggesting that Vangl2 antagonizes Dvl1's feedback inhibition. Moreover, in the same system Dvl1 induced phosphorylation of Fz3, which caused the accumulation of Fz3 in the plasma membrane and repression of the PCP pathway. This effect was antagonized by Vangl2 expression, which promoted the internalization of Fz3 and the consequent activation of the PCP pathway (Shafer et al., 2011). Since Vangl2 is highly enriched in the filopodial tips of commissural axon growth cones, the authors proposed that Wnt-stimulated growth cone turning might be mediated by the restricted antagonism of Fzd3 phosphorylation/internalization by Dvl1 and Vangl2 in filopodia (Shafer et al., 2011) (Figure 8A).

## Concluding remarks

Obviously, we are only at the beginning of understanding the signaling pathways downstream of Shh and Wnt in neural circuit formation. However, the central role of these morphogens in anteroposterior axon guidance has been clearly established in different species and in multiple neuronal populations (summarized in Tables 1, 2). As occurs during tissue morphogenesis, the Wnt and Shh signaling pathways operate simultaneously during axon guidance to ensure the fidelity of axonal projections. Often, the signaling pathways appear to act in parallel, but still with opposite activities: one gradient to “push,” the other gradient to “pull” axons in a particular direction. However, the pathways can also interact, as shown for the guidance of post-crossing commissural axons, where Shh acts directly as a repellent and indirectly by shaping an attractive Wnt activity gradient (Figure 2D; Domanitskaya et al., 2010).

**Table 1:** **Activities of Shh and Wnts in vertebrate axon guidance**.

**Ligand(s)**	**Receptor(s)**	**Model**	**Animal**	**Activity**	**References**
Shh	Boc/Smo	Pre-crossing commissural axons	Rodents	Attraction	Charron et al., 2003; Okada et al., 2006
Shh	Smo + ?	mDN axons	Mouse	Attraction	Hammond et al., 2009
Shh (low)	Ptc/Smo	RGC axons	Chick	Outgrowth	Kolpak et al., 2005; Fantetti and Fekete, 2012
		Statoacoustic ganglion axons			
Shh	Hhip + ?	Post-crossing commissural axons	Chick	Repulsion	Bourikas et al., 2005
Shh	Boc	Ipsilateral RGC axons	Rodents	Repulsion	Fabre et al., 2010
Shh (high)	Ptc/Smo	RGC axons	Chick, *Xenopus*	Repulsion	Kolpak et al., 2005; Gordon et al., 2010; Fantetti and Fekete, 2012
		Statoacoustic ganglion axons	Chick		
Shh	Ptc/Smo	Descending RST axons	Mouse	Repulsion	Song et al., 2012
Wnt4	Fz3	Post-crossing commissural axons	Rodents	Attraction	Lyuksyutova et al., 2003; Domanitskaya et al., 2010
Wnt5a/7a	?		Chick		
Wnt1, Wnt5a	Ryk	CST axons	Mouse	Repulsion	Liu et al., 2005
Wnt3	Ryk	RGC axons	Chicken	Repulsion	Schmitt et al., 2006
	Fz			Attraction	
Wnt5a	Ryk	Cortical axons	Mouse	Repulsion	Keeble et al., 2006
Wnt5a	Ryk/Fz	Cortical axons	Hamster	Repulsion	Li et al., 2009; Hutchins et al., 2011
Wnt5a	Ryk	Cortical axons	Hamster	Outgrowth	Li et al., 2009; Hutchins et al., 2011
Wnt5a	Fz3	mDN axons	Rodents	Repulsion	Blakely et al., 2011

**Table 2:** **Intracellular signaling pathways involved in axon guidance by Shh and Wnt**.

**Model**	**Ligand-receptor**	**Pathway components**	**References**
Rodent pre-crossing commissural axons	Shh/Boc/Smo	SFKs	Yam et al., 2009
Rodent post-crossing commissural axons	Shh/Cell intrinsic	14-3-3 (β, γ), via PKA	Yam et al., 2012
Chick RGC axons	Shh/Smo	PKCα, ILK	Guo et al., 2012
Chick RGC axons	Shh/Smo	Rho GTPase, nonmuscle myosin II	Kolpak et al., 2009
Mouse post-crossing commissural axons	Wnt4/Fz3	PI3K-aPKC	Wolf et al., 2008
Cortical axons	Wnt5a/Fz/Ryk	Ca^2+^, PLC, CaMKII	Li et al., 2009
5-HT and mDN neurons in mouse brainstem	Wnt5a/Wnt7b/Fz3	PCP (Fz3, Vangl2, Celsr3)	Fenstermaker et al., 2010
Mouse post-crossing commissural axons	Wnt5a/Fz3	PCP (Vangl2, Fz3, Celsr3, JNK)	Shafer et al., 2011
mDN neurons in the mouse brainstem	Wnt5a/Fz3	PCP (Fz3, Rac1)	Blakely et al., 2011

5-HT, 5-hydroxytryptamine (serotonergic); mDN, midbrain dopaminergic neuron; SFKs, Src family kinases; PKA, protein kinase A; PKC, protein kinase C; ILK, integrin-linked kinase; PI3K, phosphatidylinositol-3-kinase; PLC, phospholipase C; CaMKII, calcium/calmodulin-dependent protein kinase II; PCP, planar cell polarity pathway; JNK, c-Jun N-terminal kinase.

Although recent studies have led to the identification of several novel, non-canonical signaling pathways by which these morphogens can rapidly elicit growth cone turning, a number of key issues remain unsolved. How are the early patterning gradients that are established during tissue morphogenesis maintained or modified during later stages of neural development? Do the transcriptional pathways act in navigating neurons to modulate axon guidance responses at intermediate targets? Both Wnt and Shh are bifunctional axon guidance molecules (attractive and repulsive), but many of the signaling components that elicit these distinct signaling outputs are unknown, especially for the Wnts. How are biphasic effects of Wnts and Shh possible, such that the same axon can respond differently in a concentration-dependent manner?

Future studies will reveal whether additional cross-talk between these pathways occurs at the level of cell surface receptors, signaling modulators or intracellular signaling components. Wnt and Shh signaling may also be influenced by post-translational modifications or the contributions of non-receptor binding molecules such as heparan sulfate proteoglycans. And finally, it is also unclear whether and how morphogens interact with more “classical” axon guidance cues, such as IgSF CAMs (immunoglobulin superfamily cell adhesion molecules), Semaphorins, Netrins, or Eph/Ephrins.

### Conflict of interest statement

The authors declare that the research was conducted in the absence of any commercial or financial relationships that could be construed as a potential conflict of interest.
